# Delphinidin attenuates pathological cardiac hypertrophy via the AMPK/NOX/MAPK signaling pathway

**DOI:** 10.18632/aging.102956

**Published:** 2020-03-25

**Authors:** Youming Chen, Zhuowang Ge, Shixing Huang, Lei Zhou, Changlin Zhai, Yuhan Chen, Qiuyue Hu, Wei Cao, Yuteng Weng, Yanyan Li

**Affiliations:** 1 Department of Cardiology, Xinhua Hospital, Shanghai Jiaotong University School of Medicine, Shanghai 200092, China; 2 Department of Cardiac Surgery, Ruijin Hospital, Shanghai Jiaotong University School of Medicine, Shanghai 200025, China; 3 Department of Cardiothoracic Surgery, Tongji Hospital Affiliated to Tongji University, Shanghai 200065, China; 4 Department of Cardiology, The First Affiliated Hospital of Jiaxing University, Zhejiang 314000, China; 5 Department of Endocrinology, Xinhua Hospital, Shanghai Jiaotong University School of Medicine, Shanghai 200092, China; 6 Department of Implantology, School and Hospital of Stomatology, Tongji University, Shanghai Engineering Research Center of Tooth Restoration and Regeneration, Shanghai 200072, China

**Keywords:** delphinidin, cardiac hypertrophy, AMPK, NADPH oxidase, oxidative stress

## Abstract

Reactive oxygen species (ROS) play a pivotal role in the development of pathological cardiac hypertrophy. Delphinidin, a natural flavonoid, was reported to exert marked antioxidative effects. Therefore, we investigated whether delphinidin ameliorates pathological cardiac hypertrophy via inhibiting oxidative stress. In this study, male C57BL/6 mice were treated with DMSO or delphinidin after surgery. Neonatal rat cardiomyocytes (NRCMs) were treated with angiotensin II (Ang II) and delphinidin in vitro. Eighteen-month-old mice were administered delphinidin to investigate the effect of delphinidin on aging-related cardiac hypertrophy. Through analyses of hypertrophic cardiomyocyte growth, fibrosis and cardiac function, delphinidin was demonstrated to confer resistance to aging- and transverse aortic constriction (TAC)-induced cardiac hypertrophy in vivo and attenuate Ang II-induced cardiomyocyte hypertrophy in vitro by significantly suppressing hypertrophic growth and the deposition of fibrosis. Mechanistically, delphinidin reduced ROS accumulation upon Ang II stimulation through the direct activation of AMP-activated protein kinase (AMPK) and subsequent inhibition of the activity of Rac1 and expression of p47^phox^. In addition, excessive levels of ERK1/2, P38 and JNK1/2 phosphorylation induced by oxidative stress were abrogated by delphinidin. Delphinidin was conclusively shown to repress pathological cardiac hypertrophy by modulating oxidative stress through the AMPK/NADPH oxidase (NOX)/mitogen-activated protein kinase (MAPK) signaling pathway.

## INTRODUCTION

Heart failure is a growing public health problem [1]. Pathological cardiac hypertrophy induced by aging or mechanical and neurohormonal stimuli, such as aortic stenosis, valvular insufficiency and hypertension, is the main predisposing factor for heart failure and sudden cardiac death [2–4]. Neurohormone blockers, such as angiotensin (Ang) II receptor AT1 blockers, are used to clinically treat pathological myocardial hypertrophy and heart failure, but these drugs are not effective in reversing heart failure [5, 6]. Therefore, the need to explore the molecular mechanism of myocardial hypertrophy and develop new drugs for the treatment of heart failure is urgent.

Reactive oxygen species (ROS) are important mediators of the development of myocardial hypertrophy and heart failure [7, 8]. NADPH oxidase (NOX) is one of the main sources of myocardial ROS produced in cardiac hypertrophy [9, 10]. NOX is a multicomponent enzyme complex that consists of membrane-related NOX homologues: the p22^phox^ subunit and the cytoplasmic subunits p47^phox^, p67^phox^, p40^phox^ and the small GTP-binding protein Rac1 [11]. The small GTPase Rac1, the regulatory subunit of NOX, is a critical factor that triggers ROS production [12]. AMP-activated protein kinase (AMPK) is a major regulatory kinase that inhibits myocardial hypertrophy caused by aging and pressure overload [13, 14]. Previous studies have shown that AMPK is an important inhibitor of NOX that plays an important role in regulating antioxidant defense [15, 16]. Thus, the AMPK/NOX signaling axis may be closely related to the regulation of cardiac hypertrophy.

Given the key role of oxidative stress in the pathogenesis of cardiac hypertrophy, there is a growing interest in the use of antioxidants as a therapeutic approach. In recent years, many studies have shown that flavonoids significantly attenuate cardiac hypertrophy by inhibiting oxidant stress [17–19]. Delphinidin (2-(3,4,5-trihydroxyphenyl) chromenylium-3,5,7-triol), a flavonoid compound found in pigmented fruits and vegetables [20], possesses several biological activities, including its antioxidant [21], antiapoptotic [22], and anticancer activities [23]. Previous studies have demonstrated that the biological effects of delphinidin are mediated by the inhibition of NOX activity or the activation of AMPK [24, 25]. Therefore, delphinidin may be directly involved in regulating the AMPK/NOX signaling axis.

Despite these findings, the role of delphinidin in cardiac hypertrophy has not been reported. In addition, whether the AMPK/NOX signaling axis is involved in the mechanism by which delphinidin protects against cardiac hypertrophy remains unknown. In this study, we investigated the effect of delphinidin on aging- and pressure overload-induced cardiac hypertrophy and explored AMPK/NOX-related signaling molecules to elucidate the possible mechanism.

## RESULTS

### Molecular structure of delphinidin (Figure 1)

**Figure 1:**
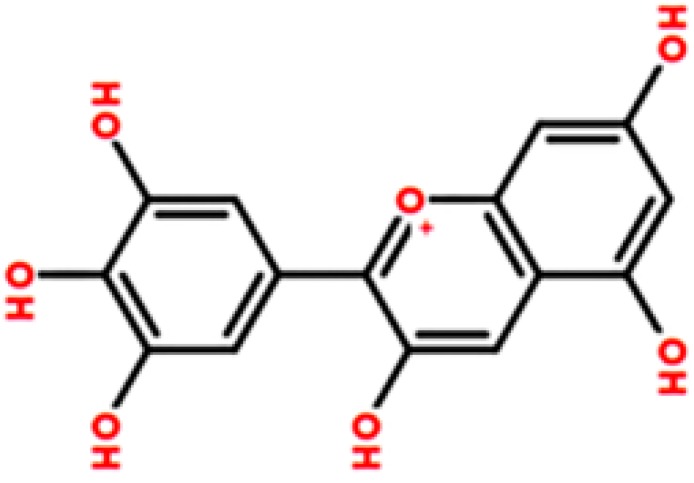
**Chemical structure of delphinidin (Dp).**

### Delphinidin ameliorated pressure overload-induced myocardial hypertrophy and oxidative stress in vivo

**Figure 2:**
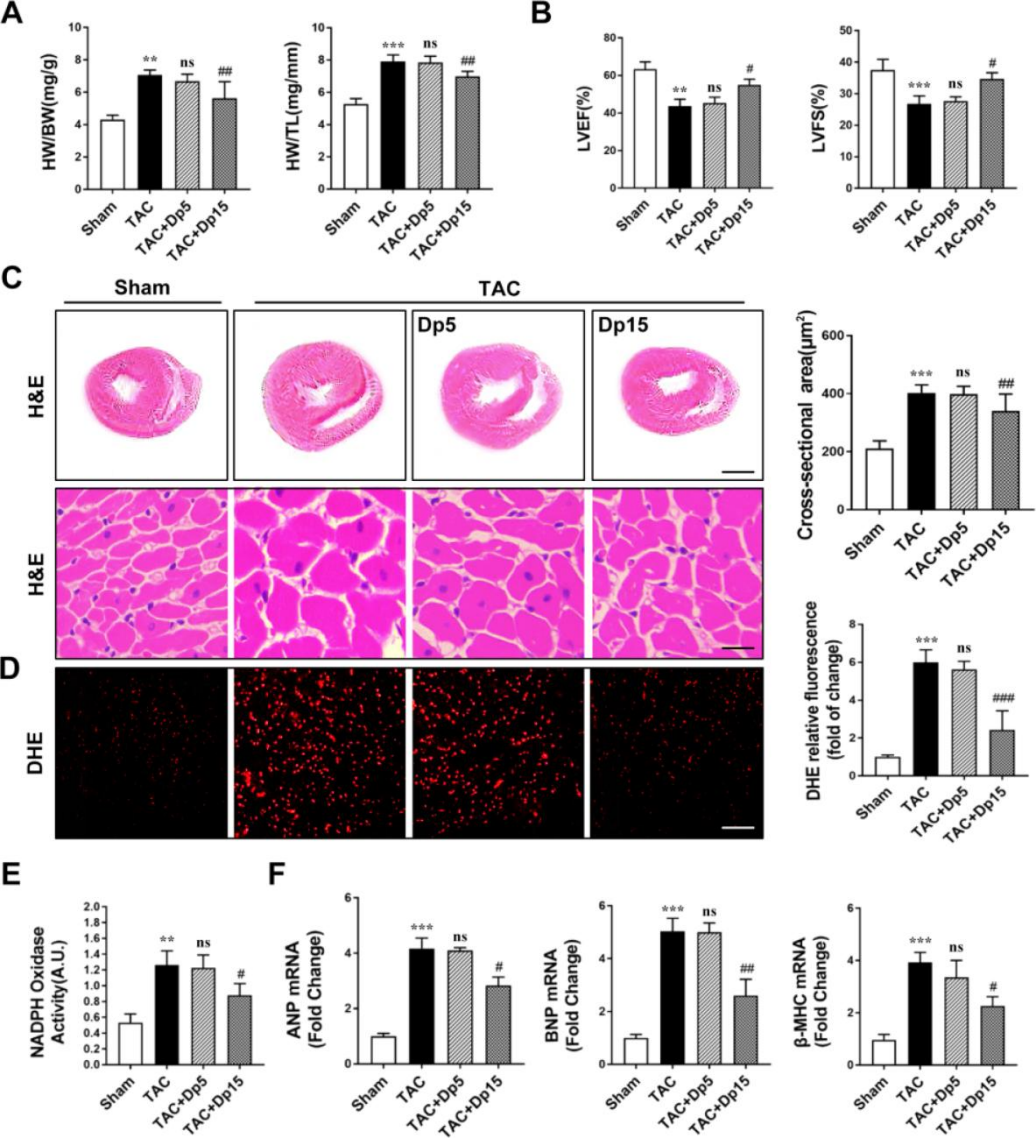
**Delphinidin attenuated cardiac hypertrophy and improved cardiac function induced by pressure overload in vivo.** (**A**) Statistical differences in the heart weight/body weight (HW/BW) and heart weight/tibia length (HW/TL) ratios between sham and TAC mice treated with vehicle or delphinidin (n=8). (**B**) Echocardiographic parameters in sham and TAC mice treated with vehicle or delphinidin (n=8). (**C**) Left, Hematoxylin-eosin (H&E) staining was performed to assess hypertrophic growth of the hearts of sham and TAC mice treated with vehicle or delphinidin (n=8). Right, Statistical analysis of differences in cardiomyocyte size (n=8). (**D**) Quantitative dihydroethidium (DHE) staining (n=8). (**E**) Chemiluminescence lucigenin assay (n=8). (**F**) Quantitative real-time PCR (qRT-PCR) was performed to analyze the mRNA levels of hypertrophic genes (n=5). In **A**–**E**, **p<0.01 versus the sham group; ***p<0.001 versus the sham group; ns versus the TAC group; #*p*<0.05 versus the TAC group; ##*p*<0.01 versus the TAC group; ###*p*<0.001 versus the TAC group. qRT-PCR was performed to analyze the mRNA levels of hypertrophic genes (n=5). In **A**–**E**, **p<0.01 versus the sham group; ***p<0.001 versus the sham group; ns versus the TAC group; #*p<0.05* versus the TAC group; ##*p<0.01* versus the TAC group; ###*p<0.001* versus the TAC group.

To determine the effect of delphinidin on cardiac hypertrophy in vivo, wild-type (WT) mice underwent TAC or sham operation; after 3 days, the mice were assigned to receive delphinidin or DMSO for 8 weeks. To verify the toxicity and side effects of delphinidin on the heart, liver, and kidney, we used a maximum dosage of 15 mg/kg/day in this study (shown in Supplementary Figure 1). Delphinidin at a dosage of 15 mg/kg/day had almost no toxic or side effects on the heart, liver, lung, and kidney. Delphinidin at the highest dosage used (15 mg/kg/day) significantly reversed TAC-induced cardiac hypertrophy, which manifested as decreased heart weight/body weight (HW/BW) and heart weight/tibia length (HW/TL) ratios (Figure 2A). In addition, the decreased left ventricular end-systolic dimension (LVESd) and left ventricular end-diastolic dimension (LVEDd) and increased left ventricular ejection fraction (LVEF) and left ventricular shortening rate (LVFS) compared with those of the sham surgery group further confirmed the effect of delphinidin at the high dosage on cardiac function (Figure 2B). Moreover, marked myocyte hypertrophy was observed at 8 weeks after surgery, as indicated by the increased cross-sectional area of the myocytes compared with that of the sham control group (hematoxylin and eosin (H&E) staining) (Figure 2C), accompanied by increased ROS levels (Figure 2D) and NOX activity (Figure 2E), but these changes were attenuated by delphinidin at the high dosage (15 mg/kg/day). However, there were no significant changes in the TAC group treated with low-dosage (5 mg/kg/day) delphinidin or DMSO. Consistently, TAC increased myocardial mRNA expression of hypertrophic markers atrial natriuretic factor (Anp), brain natriuretic peptide (Bnp), and β-myosin heavy chain (β-MHC) in WT mice, but these changes were ameliorated by delphinidin treatment at the high dosage, but not by delphinidin at the low dosage (Figure 2F). These results suggested that treatment with delphinidin at the high dosage could inhibit pathological hypertrophy, oxidative stress, and cardiac dysfunction caused by pressure overload.

### Delphinidin treatment inhibited pressure overload-induced myocardial fibrosis in vivo

**Figure 3:**
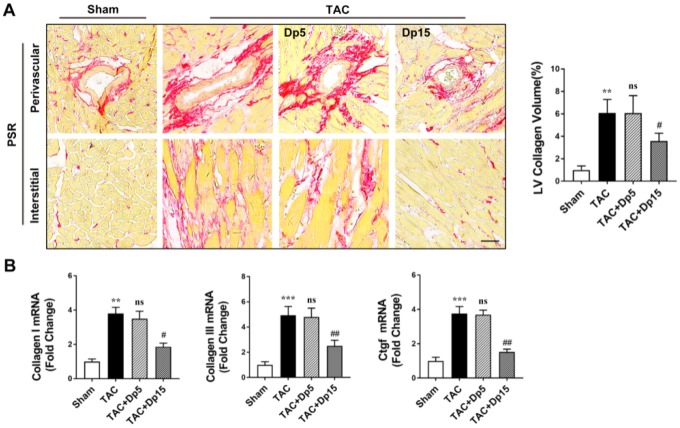
**Delphinidin attenuated pressure overload-induced myocardial fibrosis in vivo.** (**A**) Left, Representative PSR staining of histological sections of the LV (n=8). Right, Statistical analysis of differences in cardiac fibrosis. (**B**) Quantitative real-time PCR (qRT-PCR) was performed to analyze the mRNA levels of fibrosis genes (n=5). In **A**–**B**, **p<0.01 versus the sham group; ***p<0.001 versus the sham group; ns versus the TAC group; ##*p<0.01* versus the TAC group; ###*p<0.001* versus the TAC group.

We further explored the effects of delphinidin on TAC-induced myocardial fibrosis. Sustained pressure overload induced progressive interstitial fibrosis, as determined by picrosirius red (PSR) staining. TAC-triggered cardiac remodeling was abrogated, as evidenced by decreased left ventricular (LV) collagen deposition in high-dosage delphinidin-treated mice (Figure 3A). In addition, increased mRNA levels of collagen I, collagen III, and connective tissue growth factor (CTGF) in the cardiac extracellular matrix were observed in the TAC group, but these changes were dramatically abrogated after the administration of a high dosage of delphinidin (Figure 3B). However, there were no significant changes in these indices in the TAC group treated with low-dosage delphinidin or vehicle. These in vivo results suggested that delphinidin exerted a protective effect on myocardial fibrosis induced by TAC.

### Delphinidin prevented Ang II-mediated increases in oxidative stress, cellular hypertrophy, and the proliferation and activation of cultured neonatal cardiomyocytes and cardiac fibroblasts

**Figure 4:**
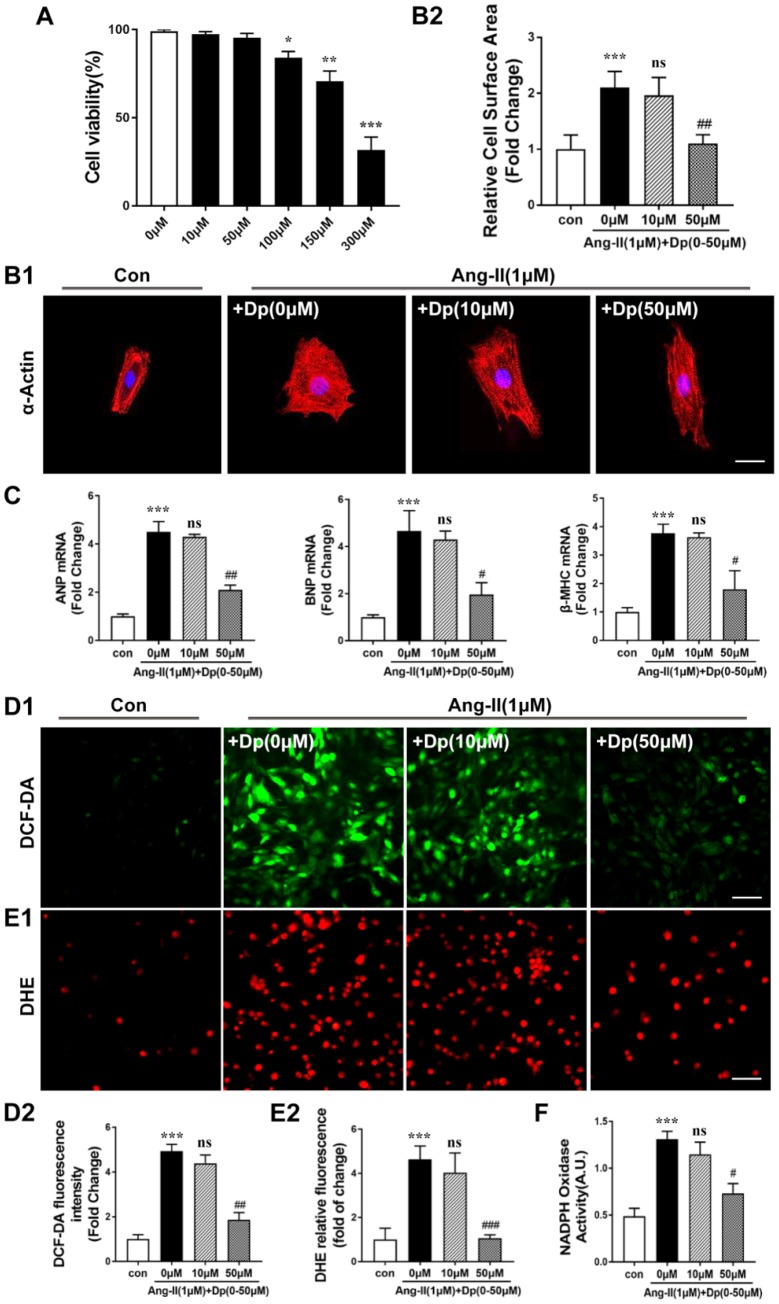
**Delphinidin inhibited Ang II-induced hypertrophy in NRCMs.** (**A**) The Cell Counting Kit-8 assay was used to detect the cell viability of cardiomyocytes treated with different concentrations of delphinidin (n=4). (**B**) NRCMs were treated with Ang II (1 μM) for 24 hours in the presence of delphinidin (10 and 50 μM) or DMSO. α-Actinin staining was performed to determine cell size. Representative images (left) and quantified cell sizes (right) of each group are shown (scale bar=20 μm). Cell surface areas (μm^2^) were measured in 3 independent experiments with at least 100 cells counted for each condition. (**C**) qRT-PCR was performed to analyze the expression of hypertrophic genes. (**D** and **E**) Representative image and results of quantitative analysis of ROS generation measured by DCF-DA and DHE staining. (**F**) Statistical analysis of differences in nicotinamide adenine dinucleotide phosphate (NADPH) oxidase activity. A.U., arbitrary units. In **A**–**F**,**p<0.01 versus the control group; ***p<0.001 versus the control group; ns versus the Ang II group; #*p<0.05* versus the Ang II group; ##*p<0.01* versus the Ang II group; ###*p<0.001* versus the Ang II group.

Cardiac hypertrophy is characterized mainly by cardiomyocyte enlargement [26]. To evaluate the cytotoxic effect of delphinidin on cardiomyocytes, we determined cell viability by CCK-8 assay. Our data showed that cardiomyocytes were viable when treated with delphinidin at concentrations below 100 μM for 24 hours (Figure 4A). To further confirm the role of delphinidin in Ang II-induced hypertrophy and oxidative stress in cardiomyocytes, we treated cardiomyocytes with Ang II (1 μM) for 24 hours after pretreatment with delphinidin at different concentrations (0, 10, 50 μM) for 30 min. The cardiac hypertrophy-related increase in cell size after Ang II stimulation was blocked by delphinidin (50 μM) (Figure 4B). Furthermore, delphinidin (50 μM) significantly prevented Ang II-induced increases in Anp, Bnp and β-MHC mRNA expression levels (Figure 4C). However, there were no significant changes in their expression following treatment with delphinidin at 10 μM for 24 hours. In addition, DHE and DCF-DA fluorescence images showed that the marked increase in ROS production in cardiomyocytes in response to Ang II stimulation was attenuated by delphinidin (50 μM) (Figure 4D and 4E). Consistent with these findings, delphinidin (50 μM) reduced the increased myocardial NOX activity after Ang II stimulation (Figure 4F). Given that cardiac fibrosis is the greatest contributor to cardiac remodeling, we further evaluated the effects of delphinidin on Ang II-mediated increases in cellular proliferation and activation in cardiac fibroblasts. Our results revealed that delphinidin significantly blocked the increase in cell proliferation induced by Ang II, judging by the results of cell counting and CCK-8 assays (Supplementary Figure 2A, 2B). Moreover, the results of scratch wound and Transwell migration assays suggested that delphinidin administration abrogated the increased cell migration induced by Ang II stimulation (Supplementary Figure 2C, 2D). Furthermore, the administration of delphinidin dramatically suppressed Ang II-induced increases in the mRNA levels of the fibrotic markers collagen I, collagen III, and CTGF (Supplementary Figure 2E). In summary, all the above results showed that delphinidin protected against pathological cell growth, oxidative stress and activation in cardiomyocytes and cardiac fibroblasts induced by Ang II in vitro.

### Delphinidin reversed pathological hypertrophy through inhibiting NOX activity by activating AMPK

**Figure 5:**
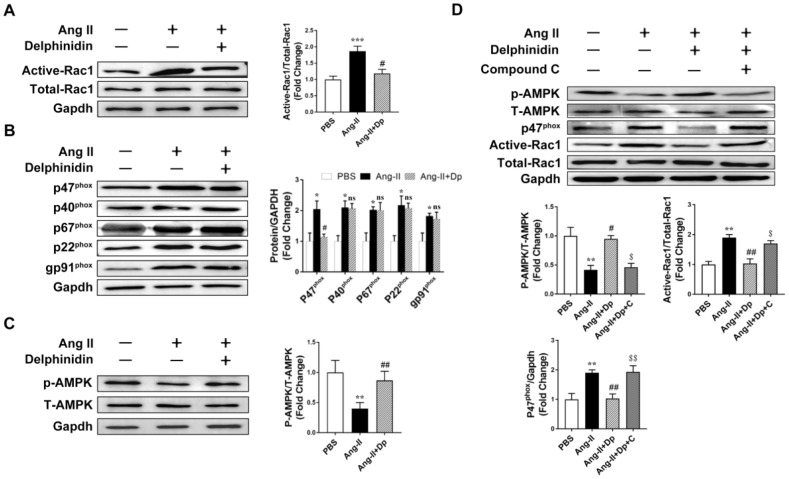
**Delphinidin downregulated NOX through activating AMPK.** (**A**) Determination of rac1 activity. Cell lysates were affinity precipitated with GTP-PBD bound to glutathione-agarose beads. Precipitated GTP-Rac1 was detected by immunoblotting with anti-Rac1 antibody (n=5). ***p<0.001 versus the PBS group; #*p<0.05* versus the Ang II group. (**B**) Expression of the NOX subunits p47^phox^, p40^phox^, p67^phox^, gp91^phox^, p22^phox^ (n=5). *p<0.05 versus the PBS group; ns versus the Ang II group; #*p<0.05* versus the Ang II group. (**C**) Representative western blot analysis revealed AMPK phosphorylation levels (n=5). **p<0.01 versus the PBS group; ##*p<0.01* versus the Ang II group. (**D**) Representative western blot analysis and GST pulldown analysis revealed the effect of delphinidin on the AMPK phosphorylation level and NADPH oxidase subunit p47^phox^ and Rac1 activity. **p<0.01 versus the PBS group; #*p<0.05* versus the Ang II group; ##*p<0.01* versus the Ang II group; $*p<0.05* versus the Ang II+Dp group; $$*p<0.01* versus the Ang II+Dp group. Bubbles and traces besides the main strips are parts of the blotting background in western blot.

**Figure 6:**
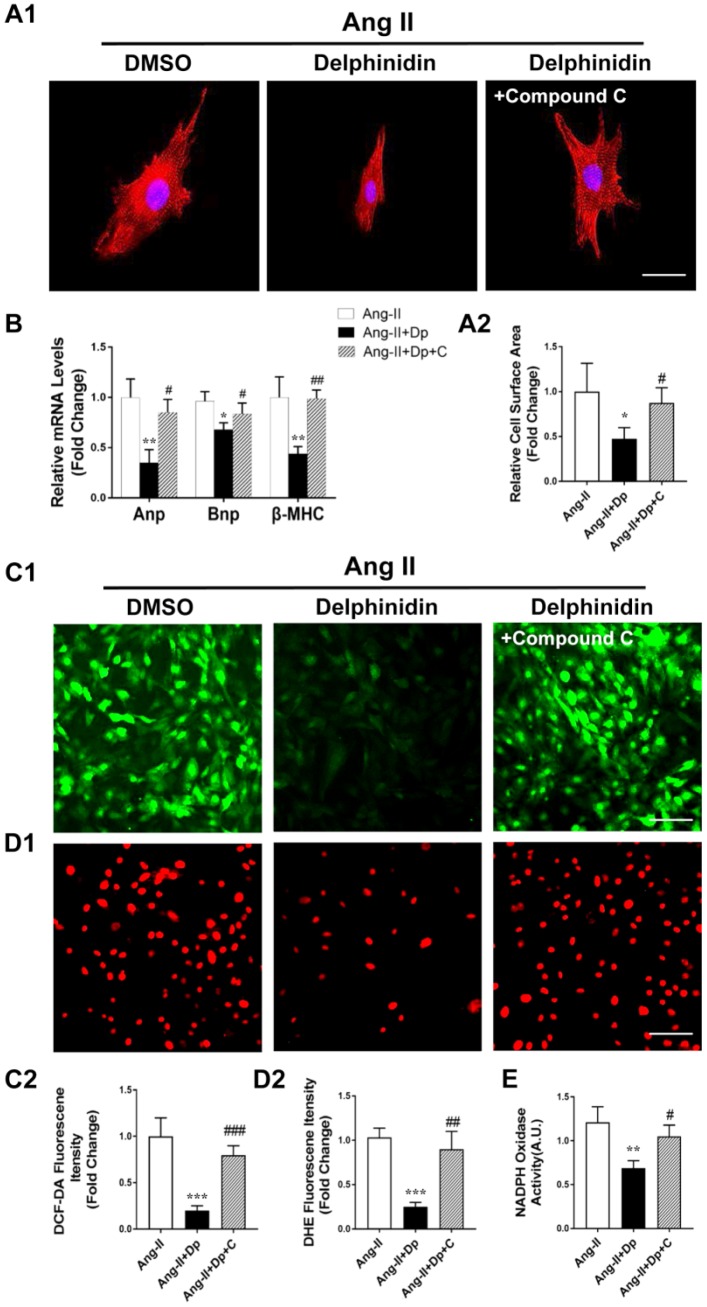
**Compound C abrogated the effects of delphinidin on Ang II-induced cardiomyocyte hypertrophy and oxidative stress by blocking AMPK activity.** (**A**) NRCMs were treated with Ang II (1 μM) for 24 hours in the presence of delphinidin (50 μM) or compound C. α-Actinin staining was performed to determine cell size. Representative images (**A1**) and quantified cell sizes (**A2**) of each group are shown (scale bar=20 μm). Cell surface areas (μm^2^) were measured in 3 independent experiments with at least 100 cells counted for each condition. (**B**) qRT-PCR was performed to analyze the expression of hypertrophic genes. (**C**, **D**), Representative image and quantitative analysis of ROS generation measured by DCF-DA and DHE staining. (**E**) Statistical analysis of differences in nicotinamide adenine dinucleotide phosphate (NADPH) oxidase activity. A.U., arbitrary units. In **A**–**E**, *p<0.05 versus the Ang II group; **p<0.01 versus the Ang II group; ***p<0.001 versus the Ang II group; #*p<0.05* versus the Ang II+Dp group; ##*p<0.01* versus the Ang II+Dp group; ###*p*<0.001 versus the Ang II+Dp group.

Delphinidin has been shown to act as an antioxidant through inhibiting NOX activity [25]. We hypothesized that delphinidin decreased Ang II-induced cardiomyocyte hypertrophy and oxidative stress by inhibiting NOX activity. A Rac1 GST-PAK pulldown assay revealed that the activation of Rac1 in cardiomyocytes was significantly increased after Ang II stimulation and that coadministration of delphinidin (50 μM) prevented this increase (Figure 5A). Immunoblot analysis showed that Ang II stimulation significantly increased protein expression levels of the NOX subunits p22^phox^, p47^phox^, p40^phox^, p67^phox^ and gp91^phox^ and that coadministration of delphinidin prevented the increases in p47^phox^ (Figure 5B). Previous studies have shown that AMPK is a physiological suppressor of NOX in multiple cardiovascular cell systems [16]. Therefore, we hypothesized that delphinidin inhibited NOX-derived ROS in cardiomyocytes by activating AMPK. Immunoblot analysis showed that treatment with delphinidin reversed the Ang II-induced decrease in AMPK phosphorylation (Figure 5C). Interestingly, the employment of an AMPK inhibitor, compound C, abolished the delphinidin-mediated decreased expression levels of p47^phox^ and increased activation of Rac1 (Figure 5D). Furthermore, compound C eliminated the delphinidin-mediated attenuation of the Ang II-induced hypertrophy of NRCMs (Figure 6A) and increased expression of pathological genes (Figure 6B), ROS production (Figure 6C and 6D) and NOX activity (Figure 6E). Similarly, compound C abolished the effects of delphinidin in alleviating TAC-induced cardiac hypertrophy and increased myocardial ROS levels and NOX activity (Supplementary Figure 3). Collectively, these results suggested that delphinidin downregulated NOX through the activation of AMPK, which suppressed ROS generation and pathological hypertrophy.

### Delphinidin regulated the MAPK signaling pathway during cardiac hypertrophy in vitro and in vivo

**Figure 7:**
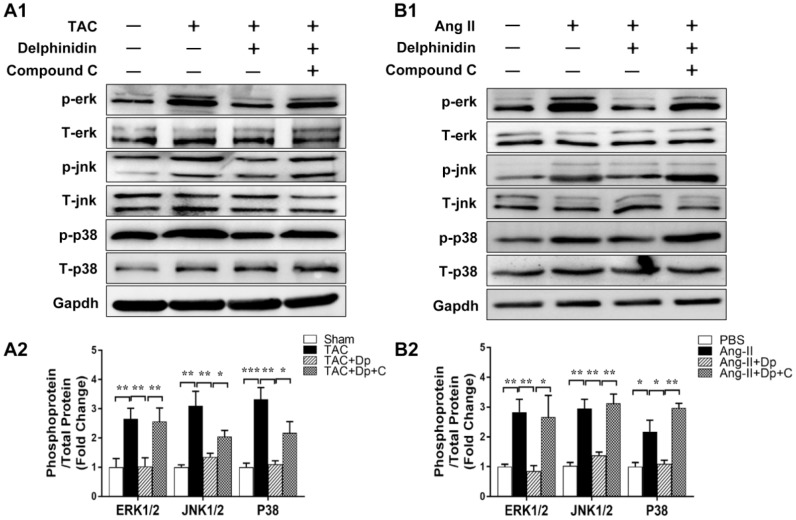
**Effect of delphinidin on the MAPK signaling pathway.** (**A1**, **B1**) Representative western blots showing total and phosphorylated ERK, JNK, and P38. (**A2**, **B2**) Quantitative results of western blot analysis (n=4); *p<0.05; **p<0.01; ***p<0.001. Bubbles and traces besides the main strips are parts of the blotting background in western blot.

To investigate the potential molecular mechanism of delphinidin in cardiac remodeling, we examined the effects of delphinidin on the mitogen-activated protein kinase (MAPK) signaling pathway. We found that phosphorylation of the kinases involved in the MAPK signaling pathway, namely, Erk1/2, Jnk1/2, and p38, was significantly increased after TAC surgery in vivo and that these changes were blocked by high-dosage delphinidin treatment (Figure 7A). We observed similar results in our in vitro experiments using Ang II-treated NRCMs (Figure 7B). Additionally, because mammalian target of rapamycin (mTOR) is another key downstream target of AMPK in the regulation of cardiac hypertrophy, we performed western blotting to assess the phosphorylation and expression of mTOR in vivo and vitro. We found that the phosphorylation level of mTOR was significantly decreased after TAC surgery. However, there was no significant difference in mTOR phosphorylation between the TAC group and TAC+ delphinidin group. We observed similar results in our in vitro experiments using Ang II-treated NRCMs (Supplementary Figure 4). Collectively, these data suggested that MAPK signaling regulation is responsible for the antihypertrophic effects of delphinidin.

### Delphinidin suppressed cardiac hypertrophy in aged mice

**Figure 8:**
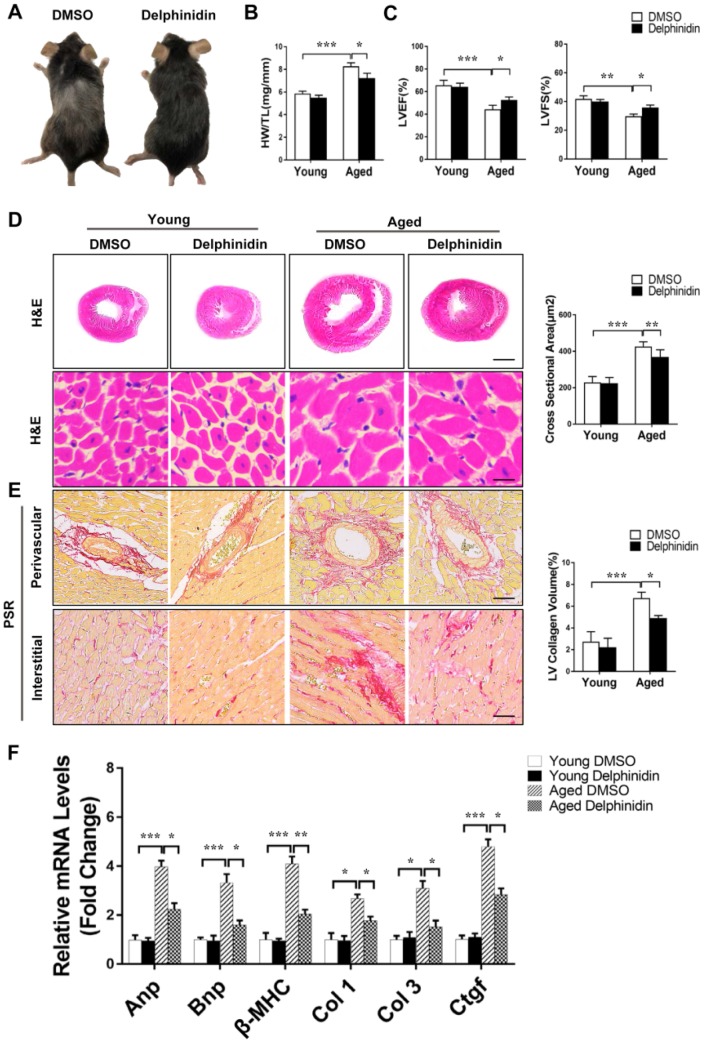
**Delphinidin reduced cardiac hypertrophy in aged mice.** (**A**) Representative gross morphology of young and aged mice administered delphinidin and DMSO. (**B**) Statistical analysis of differences in the heart weight/tibia length (HW/TL) ratio (n=6). (**C**) Left ventricular ejection fraction and fractional shortening of young and aged mice administered delphinidin and DMSO (n=6). (**D**) Left, H&E staining was performed to assess hypertrophic growth of the hearts of young and aged mice administered with delphinidin and DMSO. Right, Statistical analysis of differences in cardiomyocyte size (n=6). (**E**) Left, Representative PSR staining of histological sections of the LV (n=6). Right, Statistical analysis of differences in cardiac fibrosis. (**F**) Quantitative real-time PCR (qRT-PCR) was performed to analyze the mRNA levels of hypertrophic genes and fibrosis genes (n=5). In **B**–**F**, *p<0.05, **p<0.01, ***p<0.001.

**Figure 9:**
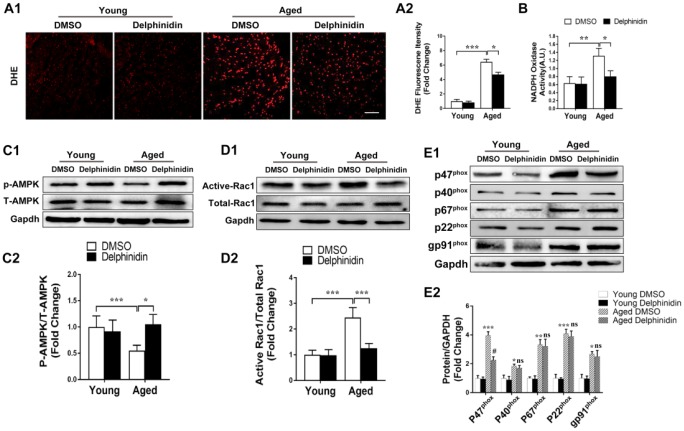
**Delphinidin reduced ROS production in the aged myocardium through inhibiting NOX by activating AMPK.** (**A**) Quantitative dihydroethidium (DHE) staining (n=8). (**B**) Statistical analysis of differences in nicotinamide adenine dinucleotide phosphate (NADPH) oxidase activity. A.U., arbitrary units. (**C**) Representative western blot analysis revealed AMPK phosphorylation levels (n=4). (**D**) Determination of rac1 activity. The cell lysates were affinity precipitated with GTP-PBD bound to glutathione-agarose beads. Precipitated GTP-Rac1 was detected by immunoblotting with anti-Rac1 antibody (n=4). (**E**) Expression of the NOX subunits p47^phox^, p40^phox^, p67^phox^, gp91^phox^, and p22^phox^ (n=4). In **A**–**D**, *p<0.05, **p<0.01, ***p<0.001. In (**E**) *p<0.05 versus the young+DMSO group; **p<0.01 versus the young+DMSO group; ***p<0.001 versus the young+DMSO group; ns versus the aged+DMSO group; #*p*<0.05 versus the aged+DMSO group.

**Figure 10:**
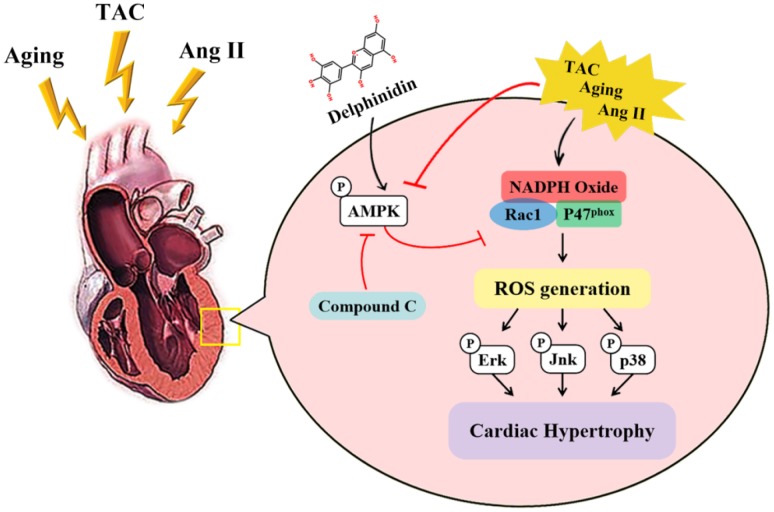
**Cartoon demonstrating that delphinidin attenuates pathological cardiac hypertrophy via the AMPK/NOX/MAPK signaling pathway.**

To investigate the effect of delphinidin on aging-related cardiac hypertrophy, 4-month-old and 18-month-old mice were administered delphinidin (15 mg/kg/day) or DMSO for 6 months. Remarkably, aged mice administered delphinidin showed less of the visible characteristics of aging (e.g. hair browning) than aged mice administered DMSO, indicating that delphinidin might affect the health of aged mice (Figure 8A). Young and aged mice administered delphinidin or DMSO were subjected to functional cardiac phenotyping. We found that delphinidin significantly attenuated aging-related cardiac hypertrophy, as supported by of the observed HW/TL ratios (Figure 8B). Delphinidin significantly reduced echocardiography-detected dysfunction in aged mice, as evidenced by increases in the LVEF and LVFS (Figure 8C). However, there were no significant changes in young mice administered delphinidin or DMSO. Consistent with these findings, pathological myocardial remodeling was significantly attenuated in aged mice administered delphinidin compared with aged mice administered DMSO, as shown by marked decreases in the size of the cardiomyocytes (Figure 8D) and cardiac fibrosis (Figure 8E). Moreover, the increased expression of hypertrophy markers (Anp, Bnp, and β-MHC) and fibrosis markers (collagen I, collagen III, and Ctgf) was dramatically abrogated in aged mice administered delphinidin (Figure 8F). Finally, we explored the mechanism by which delphinidin reduced aging- induced myocardial hypertrophy. Previous studies have shown that aging-dependent cardiac hypertrophy is closely related to ROS production [27]. Therefore, ROS production in the hearts of young and aged mice administered delphinidin and DMSO was analyzed. Quantitative DHE staining and a chemiluminescence lucigenin assay revealed a marked decrease in superoxide production and NOX activity in aged mice administered delphinidin compared with aged mice administered DMSO (Figure 9A, 9B). We further explored whether the AMPK/NOX signaling axis was involved in the mechanism by which delphinidin protected against aging-related cardiac hypertrophy. We found that AMPK phosphorylation was significantly increased and that the expression of p47^phox^ and the activity of Rac1 were decreased in aged mice administered delphinidin compared with aged mice administered DMSO. However, there were no significant changes in the expression of p22phox, p67^phox^, p40^phox^, or gp91^phox^ (Figure 9C–9E). These findings suggested that delphinidin significantly reduced ROS production in the aged myocardium by inhibiting the phosphorylation of AMPK and the activity of NOX. Delphinidin significantly reversed aging-related cardiac hypertrophy. Delphinidin attenuates pathological cardiac hypertrophy via the AMPK/NOX/MAPK signaling pathway (Figure 10).

### DISCUSSION

In this study, we investigated the role of delphinidin in myocardial hypertrophy induced by aging and pressure overload in vivo and in Ang II-induced cardiomyocyte hypertrophy in vitro. The results showed that delphinidin significantly inhibited pathological cardiomyocyte hypertrophy and cardiac fibrosis. Mechanistic experiments demonstrated that delphinidin repressed the expression of p47^phox^ and the activity of Rac1 through the activation of AMPK. These changes were achieved by suppressing the MAPK signal transduction pathway. To the best of our knowledge, this study has demonstrated for the first time that delphinidin plays an important inhibitory role in the progression of pathological myocardial hypertrophy and fibrosis via the AMPK/NOX/MAPK pathway.

NOX-derived ROS play an important role in the development and progression of cardiac hypertrophy [28, 29]. The cytoplasmic regulatory subunits of NADPH (p40^phox^, p47^phox^, p67^phox^ and Rac1) are transferred to the plasma membrane for oxidase activation and ROS production in the process of pathological cardiac hypertrophy [30]. When the activity of NOX was blocked, the production of ROS was significantly reduced, and myocardial hypertrophy was inhibited [31, 32]. Previous studies showed that many flavonoids, including puerarin-7-O-glucuronide [33] and troxerutin [34], significantly inhibited cardiomyocyte hypertrophy by blocking the activity of NOX. Delphinidin, an anthocyanidin, has been reported to have potent antioxidant and anti-inflammatory activities [35, 36]. In this study, we discovered that delphinidin significantly reduced Ang II- and aging-induced oxidative damage by inhibiting the activity of NOX. The sources of ROS in cardiomyocytes include mitochondria, lipoxygenase, cyclooxygenase and xanthine oxidase. We confirmed only that delphinidin significantly reduced NOX-mediated ROS production. Whether delphinidin has a similar effect on ROS production by mitochondria or other sources needs further exploration.

Although delphinidin played a vital role in reducing ROS generation in Ang II-induced cardiomyocyte hypertrophy, it could not directly regulate the activity of NOX. Our report shows that delphinidin inhibited pathological cardiac hypertrophy though directly regulating the activity of AMPK. AMPK, a major regulatory kinase containing one catalytic subunit (α) and two regulatory subunits (β and γ), is an important regulator of metabolism, but it also plays a pivotal role in maintaining redox balance [37]. AMPK is a physiological inhibitor of NOX in the cardiovascular system [16, 38] and central nervous system [39]. Our current data demonstrated that pretreatment with compound C, a selective AMPK inhibitor, abolished the suppressive effects of delphinidin on Ang II-induced upregulation of p47^phox^ and the activation of Rac1 and antagonized the delphinidin-mediated inhibition of Ang II-induced cardiomyocyte hypertrophy, suggesting that the protective effect of delphinidin is due, at least in part, to its ability to upregulate the activation of AMPK.

In addition to causing molecular damage, ROS regulate multiple molecular signaling pathways related to cardiac hypertrophy. The current study demonstrated that ROS production is the main player triggering the MAPK signaling pathway, a classical pathway involved in oxidative stress-induced hypertrophy [40, 41]. Previous studies have shown that delphinidin plays a biological role by inhibiting the MAPK signaling pathway [42–44]. In line with the above findings, our data demonstrated that delphinidin markedly inhibited the activation of ERK1/2, p38 and JNK1/2 in vivo and in vitro, suggesting that delphinidin inhibits cardiac hypertrophy by blocking ROS-dependent MAPK signaling. Interestingly, it has been demonstrated that many flavonoids, such as puerarin [19], hesperetin [45] and taxifolin [17], also repress oxidative stress-mediated cardiomyocyte hypertrophy through inhibiting the MAPK signaling pathway. Therefore, we believe that flavonoids play a common role in inhibiting myocardial hypertrophy by blocking the ROS/MAPK signaling pathway.

Furthermore, we explored whether delphinidin reduces aging-associated cardiac hypertrophy. Aging is one of the main risk factors for cardiac hypertrophy [46]. The hallmarks of cardiac aging include cardiomyocyte senescence, fibroblast proliferation, inflammation, and hypertrophy. Imbalance between the levels of ROS and antioxidant enzymes is greatly enhanced in aging cells, promoting cardiac remodeling [47]. According to our findings, elevated AMPK activity and downregulated NOX expression were observed in the hearts of aging mice compared with young littermates, and delphinidin reversed these phenotypes and improved cardiac function in aging mice. Thus, daily consumption of delphinidin could possibly preserve heart function affected by the detrimental effects of aging.

Notably, clinical data show that delphinidin may exhibit health-promoting effects by ameliorating oxidative stress. Delphinidin is one of the major anthocyanidins found in nature. Delphinol, an extract of maqui berries containing 35% total anthocyanins and 28% total delphinidin, was proven to reduce the levels of ox-LDL and urinary F2-isoprostanes and ameliorate altered oxidative status in healthy adults, overweight adults, and adult smokers by a double-blind, placebo-controlled study [48]. In another study, the regular consumption of delphinol (180 mg a day before breakfast for three months) was suggested to improve blood sugar and blood lipids in subjects with early prediabetes [49]. As shown by the oral glucose tolerance test (OGTT), delphinol (60, 120, and 180 mg/day) dosage-dependently lowered basal glycaemia and insulinemia in prediabetic subjects [50].

Taken together, these results provide the first evidence that delphinidin attenuates pathological myocardial hypertrophy via blocking oxidative stress by activating AMPK and inhibiting the NOX/MAPK signaling pathway and suggest an effective approach for preventing cardiac hypertrophy and heart failure.

## MATERIALS AND METHODS

### Animals and treatments

Eight-week-old male C57BL/6J mice were purchased from Vital River (Beijing, China). The experimental procedures were approved by the Animal Care and Use Committee of Shanghai Xinhua Hospital affiliated with the Shanghai Jiao Tong University School of Medicine. The mice were subjected to transverse aortic constriction (TAC)-induced pressure overload according to a previous study [51]. Three days after TAC or sham operation, the animals were treated daily with the same volume of vehicle (DMSO) or 5 or 15 mg/kg delphinidin dissolved in vehicle (DMSO) (5 and 15 mg/kg body weight/day) and the intraperitoneal injection of 20 mg/kg compound C (an AMPK inhibitor) for 8 weeks. The concentration of delphinidin employed in this experiment was in accordance with those used in human beings according to the National Health and Nutrition Inspection Survey [52].

### Reagents

The delphinidin used to treat cells and animals was purchased from ChromaDex (Los Angeles, USA). The compound C used to treat cells and animals was purchased from Macklin (Shanghai, China). An ROS assay kit (dihydroethidium (DHE) and 2′-7′-dichlorofluorescein diacetate (DCFH-DA)) was obtained from BestBio (Shanghai, China). An Rac1 activation assay kit was obtained from Millipore (Temecula, CA, USA). Dulbecco’s modified Eagle’s medium (DMEM), fetal bovine serum (FBS) and collagenase type II were purchased from Gibco (Los Angeles, CA, USA). Fluorescent secondary antibodies were purchased from Thermo Scientific (Life Technologies, USA). Antibodies against p67^phox^, p47^phox^, p40^phox^, gp91^phox^, p22^phox^, phospho-AMPKα (Thr^172^), AMPKα, phospho-ERK1/2 (4370) at Thr^202^/Tyr^204^, ERK1/2 (4695), phospho-P38 (4511) at Thr^180^/Tyr^182^, P38 (9212), phospho-JNK1/2 (4668) at Thr^183^/Tyr^185^ and JNK1/2 (9258) were purchased from Cell Signaling Technology. Lysis buffer, anti-Rac1 antibody, anti-GAPDH antibody, a BCA protein assay kit and a NOX activity kit were purchased from Beyotime Biotechnology (Shanghai, China). All other reagents were purchased from Epizyme (Shanghai, China).

### Primary culture of neonatal rat cardiomyocytes and experimental treatments

Neonatal rat cardiomyocytes (NRCMs) were isolated from 1- to 2-day-old Sprague–Dawley rats as previously described [13]. After isolation, the cardiomyocytes were plated on 0.1% gelatin-coated dishes in the presence of 10% serum. After 24 hours, the NRCMs were maintained in serum-free DMEM for 12 hours. Then, the cells were exposed to Ang II (1 μM) and delphinidin at different concentrations for 24 hours. Compound C (10 μM) was applied to the medium 2 hours before Ang II and delphinidin treatment.

### Determination of Rac1 activity

The cardiac tissue and cultured cardiomyocytes were washed with phosphate-buffered saline (PBS) 3 times. Proteins were extracted from cardiac tissue and cardiomyocytes with cleavage buffer (formula: 50 mM Tris-HCl, pH 7.5, 1% NP-40, 100 mM NaCl, 10% glycerol, 10 mM MgCl_2_ and protease inhibitor) on ice for 40 min. The cell homogenates were centrifuged at 13,000 rpm for 10 min. The supernatant (100 μl) was used to determine the total amount of Rac1, and the remaining supernatant was incubated with 20 μg of GST-PAK-CRIB domain (PAK-CD) for 1 hour (the GST-PAK-CRIB fusion protein was coupled to glutathione-agarose beads.). Agarose beads coupled with active Rac1 were collected by centrifugation. The amount of active Rac1 was determined by western blotting.

### Measurement of NOX activity and evaluation of oxidative stress

The activity of NOX was determined by the lucigenin chemiluminescence method. Briefly, homogenates of cardiac tissues and cardiomyocytes were collected in PBS containing protease inhibitor and phosphatase inhibitor and then centrifuged at 1000 ×g for 10 min. The supernatants were then collected, and lucigenin (50 μM) and NADPH (1 mM) were added for the NOX activity assay. Data were calculated as the change in the rate of luminescence per minute per milligram of protein.

The production of ROS in the ventricular sections of TAC- and sham-operated mice was evaluated by observing the red fluorescence intensity following DHE (10 μM) staining. Briefly, frozen heart tissue was cut into 4 μm sections, which were incubated with DHE in PBS for 40 min at 37 °C in a humidified chamber in the dark. Similarly, intracellular superoxide anions were determined with the fluorescent probes DHE and DCF-DA. After the experiment, the cardiomyocytes were washed with cold DPBS three times. The cardiomyocytes were incubated with DHE (10 μM) and DCF-DA (10 μM) at 37 °C for 30 min. After washing with cold DPBS twice to remove free DHE and DCF-DA, images of the cardiomyocytes were captured and analyzed under an inverted fluorescence microscope (Olympus IX71, Japan).

### Transwell migration, CCK-8 and scratch wound assays

Transwell migration, CCK-8 and scratch wound assays were performed as previously described [53].

### Quantitative real-time PCR

RNA was extracted from cardiomyocytes and mouse ventricles by using TRIzol reagent (TaKaRa, Kyoto, Japan) and reverse transcribed into cDNA using the PrimeScriptTM RT Reagent Kit (TaKaRa). RT-qPCR was carried out using SYBR Green (TaKaRa), and the results were normalized to GAPDH expression. All primer sequences are shown in Supplementary Table 1.

### Western blot analysis

Total protein was extracted from frozen left ventricular tissues and NRCMs using lysis buffer. The protein concentrations were measured using a BCA assay kit (Pierce) according to the manufacturer’s instructions. The proteins (20-40 μg) were separated using SDS-PAGE and then transferred to PVDF membranes. The membranes were blocked in 5% nonfat milk in TBST and then incubated with diluted primary antibodies (1:1000) overnight at 4°C. After washing with TBST, the membranes were incubated with horseradish peroxidase-conjugated secondary antibody for 1 hour at room temperature. Finally, the protein signals were detected with a Bio-Rad ChemiDocTM XRS+ system (Bio-Rad).

### Statistical analysis

The data are presented as the *means ± SDs*. Statistical analyses of comparisons between two groups or multiple groups were performed with Student’s t-test and multiple-comparison analysis of variance (ANOVA), respectively. A value of *P<0.05* indicated a statistically significant difference.

## Supplementary Material

Supplementary Figures

Supplementary Table 1
