# Supplementary material for: Delphinidin attenuates pathological cardiac hypertrophy via the AMPK/NOX/MAPK signaling pathway
Source: Aging (Albany NY). 2020 Mar 25;12(6):5362–83. doi: 10.18632/aging.102956 (PMC7138591; doi:10.18632/aging.102956)
Supplement: Supplementary Figures [file aging-12-102956-s002..pdf]

SUPPLEMENTARY FIGURES

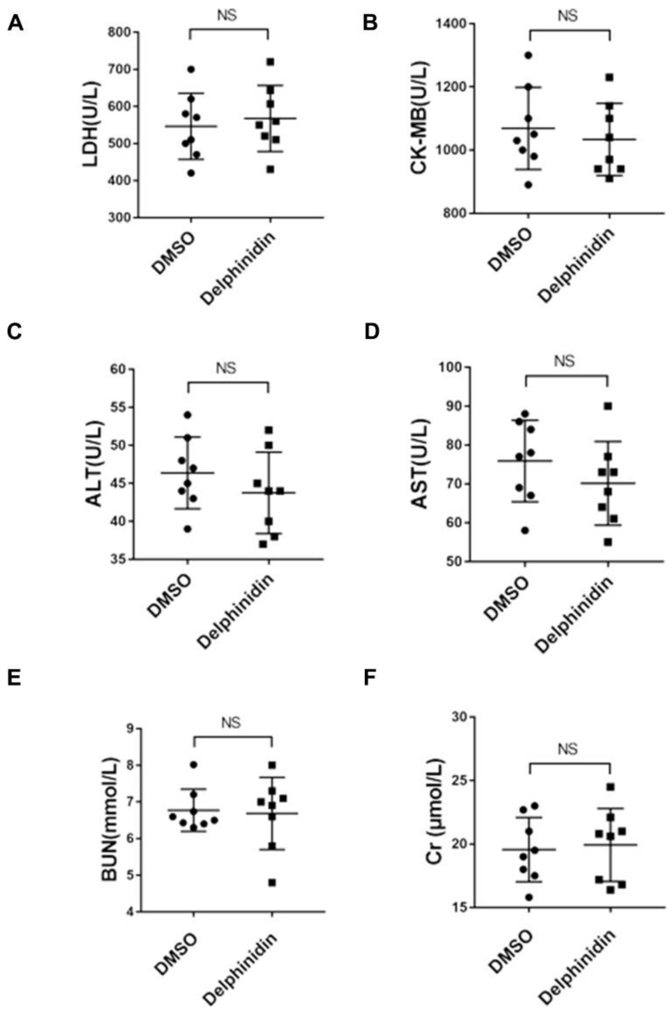

Supplementary Figure 1. Verification of the toxicity of 15 mg/kg/day delphinidin and its side effects on the heart, liver and kidney. (**A**, **B**) Detection of the levels of the serum myocardial enzymes LDH and CK-MB. (**C**, **D**) Detection of the levels of the serum liver function indicators AST and ALT. (**E**, **F**) Detection of the levels of the serum renal function indicators BUN and Cr. LDH=lactate dehydrogenase; CK-MB=creatine kinase isoenzymes; AST=glutamic oxaloacetic transaminase; ALT=alanine aminotransferase.

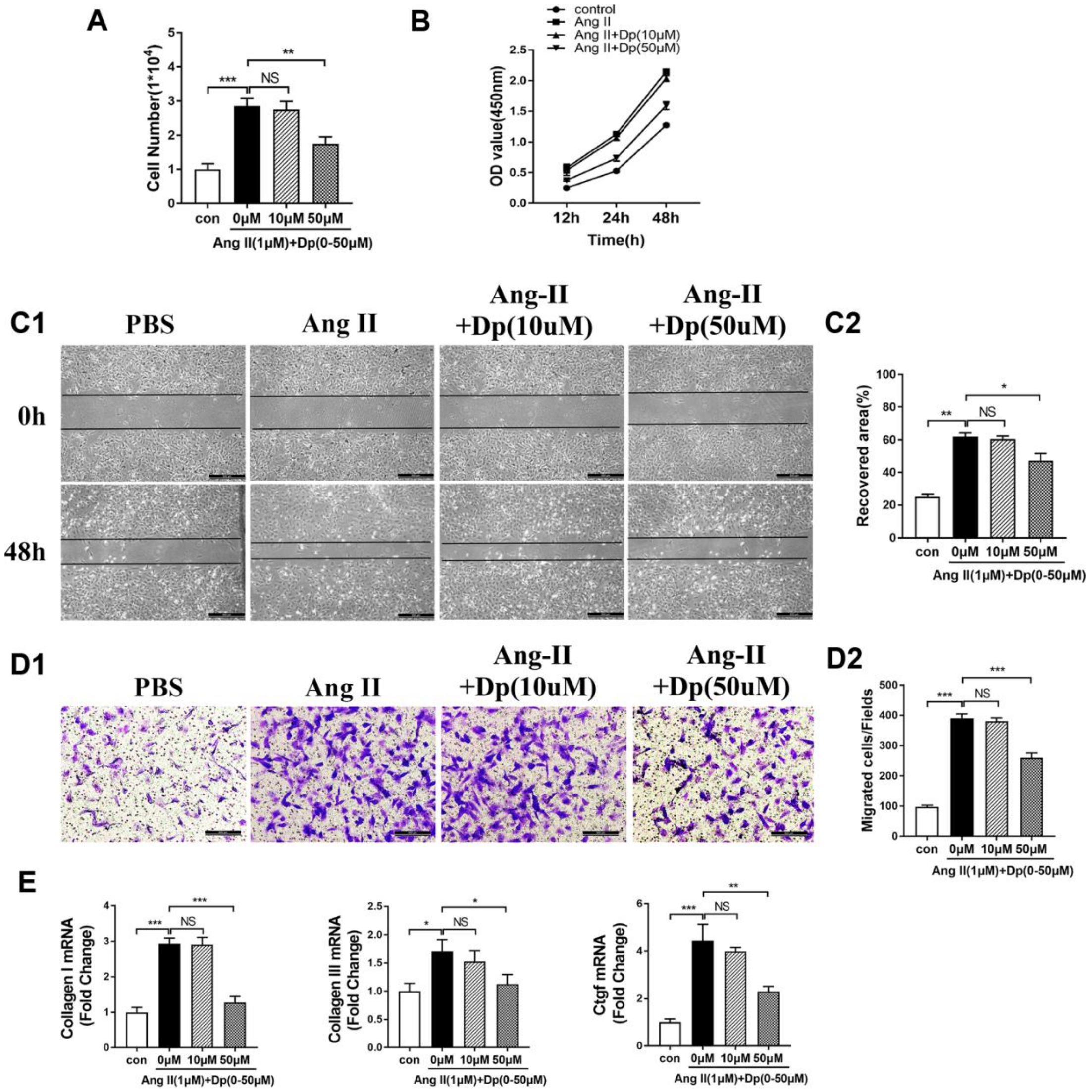

Supplementary Figure 2. Delphinidin ameliorated cardiac fibrosis induced by Ang II in vitro. (**A**, **B**) Quantification of cells by cell counting and results of the CCK-8 assay (n=4 in each group). (**C**) Scratch wound assays were performed to assess the migratory capacities of cells in the indicated groups (n=4 in each group; scale bar=500 μm). (**D**) Representative images from the Transwell migration assay and quantification of migrated fibroblasts of the indicated groups (n=4 in each group; scale bar=100 μm). (**E**) mRNA levels of collagen I, collagen III, and CTGF determined by qRT-PCR (n=4 in each group). In **A**–**E**, *$p<0.05$, **$p<0.01$ and ***$p<0.001$ between the two indicated groups.

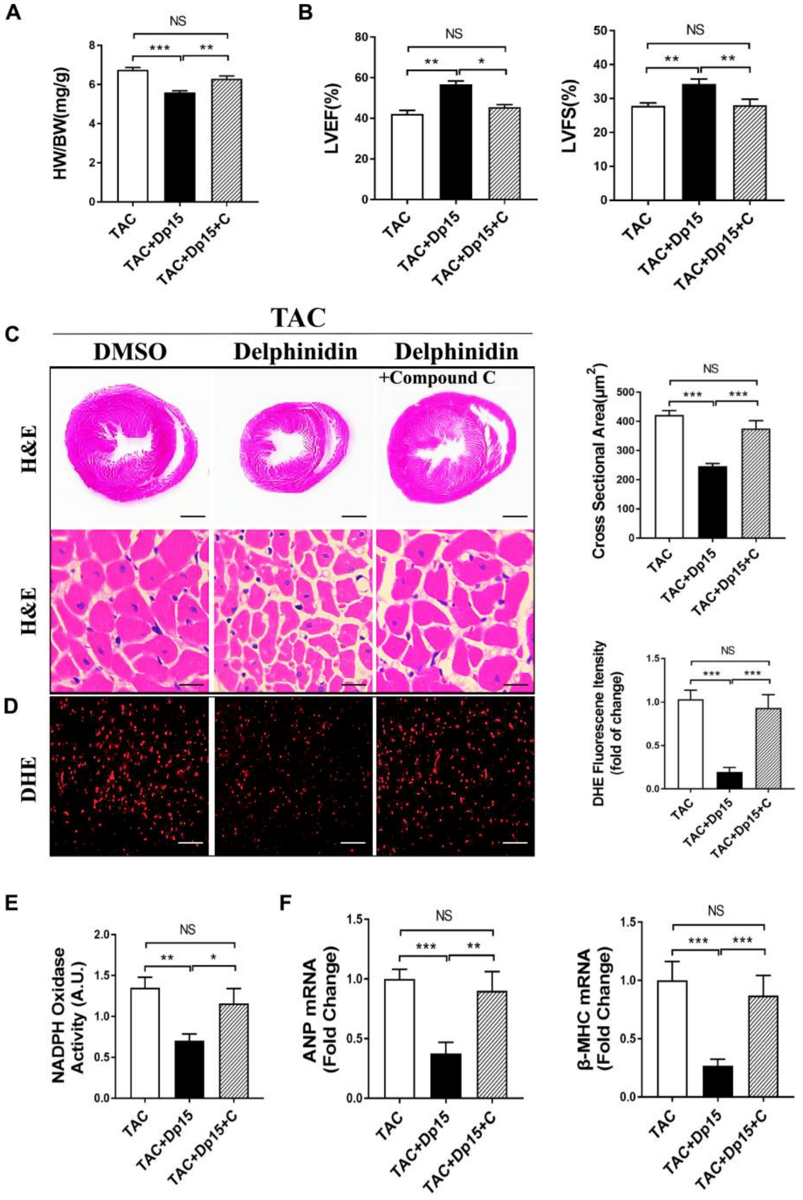

Supplementary Figure 3. Compound C abolished delphinidin-mediated alleviation of TAC-induced cardiac hypertrophy and oxidative stress. (**A**) Statistical analysis of differences in the HW/BW and HW/TL ratios (n=8). (**B**) Echocardiographic parameters in sham and TAC mice treated with vehicle or delphinidin (n=8). (**C**) Left, Hematoxylin-eosin (H&E) staining was performed to assess hypertrophic growth of the hearts of sham and TAC mice treated with vehicle or delphinidin (n=8). Right, Statistical analysis of differences in cardiomyocyte size (n=8). (**D**) Quantitative dihydroethidium (DHE) staining (n=8). (**E**) Chemiluminescence lucigenin assay (n=8). (**F**) Quantitative real-time PCR (qRT-PCR) was performed to analyze the mRNA levels of hypertrophic genes (n=5). In **A**–**F**, **p<0.05,* ***p<0.01* and ****p<0.001* between the two indicated groups.

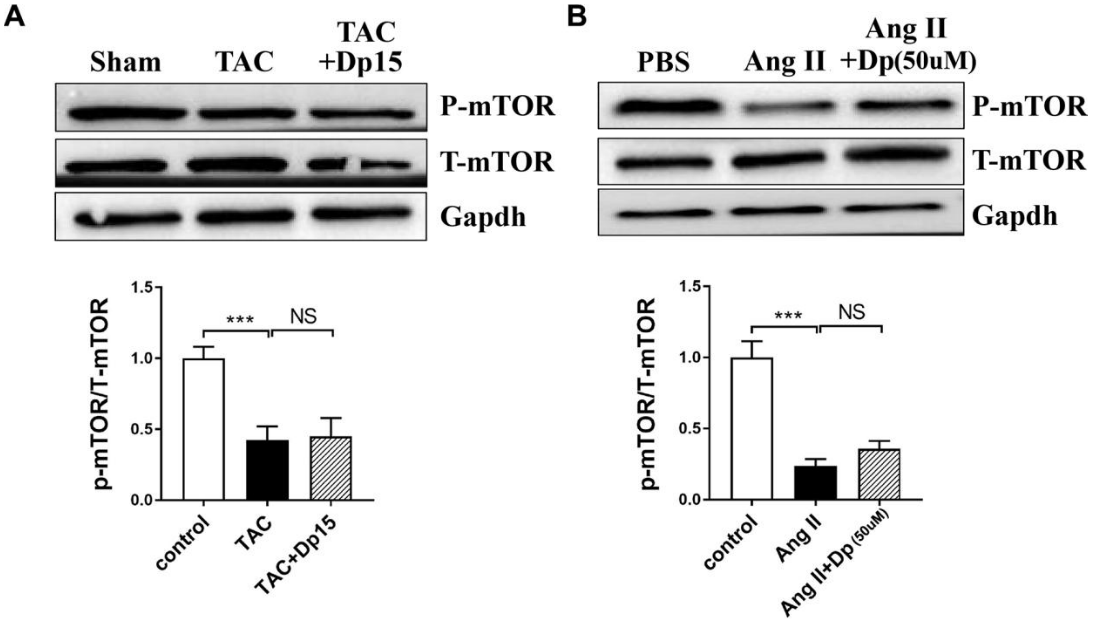

Supplementary Figure 4. mTOR did not contribute to the delphinidin-mediated reduction in pathological cardiac hypertrophy. (**A**) Western blot analysis of mTOR in cardiac tissues from the indicated groups (n=3). (**B**) Western blot analysis of mTOR in cardiomyocytes from the indicated groups (n=3). ****p*<0.001, NS indicates no significant difference between the two indicated groups.
