# Supplementary material for: Delphinidin attenuates pathological cardiac hypertrophy via the AMPK/NOX/MAPK signaling pathway
Source: Aging (Albany NY). 2020 Mar 25;12(6):5362–83. doi: 10.18632/aging.102956 (PMC7138591; doi:10.18632/aging.102956)
Supplement: Supplementary Table 1 [file aging-12-102956-s001..pdf]

SUPPLEMENTARY TABLE

Supplementary Table 1. The primer sequence for qRT-PCR.

Gene	Forward primer	Reverse primer
Mouse-ANP	5′-ACCTGCTAGACCACCTGGAG-3′	5′-CCTTGGCTGTTATCTTCGGTACCGG-3′
Mouse-β-MHC	5′-CCGAGTCCCAGGTCAACAA-3′	5′-CTTCACGGGCACCCTTGGA-3′
Mouse-GAPDH	5′-ACTCCACTCACGGCAAATT C-3′	5′-TCTCCATGGTGGTGAAGACA-3′
Mouse-BNP	5′-GAGGTCACTCCTATCCTCT-3′	5′-GCCATTTCCTCCGACTTTTCTC-3′
Mouse-Collagen-1	5′-AGGCTTCAGTGGTTTGGATG-3′	5′-CACCAACAGCACCATCGTTA-3′
Mouse-Collagen-lll	5′-CCCAACCCAGAGATCCCATT-3′	5′-GAAGCACAGGAGCAGGTGTAGA-3′
Mouse-CTGF	5′-TGACCCCTGCGACCCACA-3′	5′-TACACCGACCCACCGAAGACACAG-3′
Rat-ANP	5′-TGAGCGAGCAGACCGATGAAGC-3′	5′-GCCGCGCCCGAGAGCAC-3′
Rat-β-MHC	5′-TGAAGAGCACATGGCCACCGATAG	5′-ATGGCGCCTGTCAGCTTGTAAATG-3′
Rat-BNP	5′-CAGCAGCTTCTGCATCGTGGAT-3′	5′-TTCCTTAATCTGTCGCCGCTGG-3′
Rat-GAPDH	5′-CATGGCCTTCCGTGTTCCTACCC-3′	5′-GCCGCCTGCTTCACCACCTTCT-3′
